# Interdisciplinary Rendez-Vous Approach in Endovascular Stroke Treatment: A New Concept to Accelerate Mechanical Thrombectomy in Primary Stroke Centers

**DOI:** 10.1007/s00270-023-03610-y

**Published:** 2023-11-21

**Authors:** Nadja Krug, Holger Braun, Andreas Knez, Holger Auerbach, Stephen Bodenberger, Bettina Eglseder, Jan Kirschke, Tobias Boeckh-Behrens, Silke Wunderlich, Julia Henninger, Sandra Boy, Martin Renz, Dominik Sepp, Claus Zimmer, Christian Maegerlein

**Affiliations:** 1 Diagnostic and Interventional Neuroradiology, University Hospital Basel, Basel, Switzerland; 2 Medical Department, Krankenhaus Weilheim, Weilheim, Germany; 3 Department of Diagnostic and Interventional Neuroradiology, Klinikum Rechts der Isar, School of Medicine, Technical University Munich, Munich, Germany; 4 Department of Neurology, Klinikum Rechts der Isar, School of Medicine, Technical University of Munich, Munich, Germany; 5 Medical Department, Klinikum Dritter Orden, Munich, Germany; 6 Department of Neurology, Asklepios Stadtklinik Bad Tölz, Bad Tölz, Germany

**Keywords:** Mechanical Thrombectomy, Stroke, Primary Stroke Center, Endovascular Stroke Treatment, Rendez-Vous concept

## Abstract

**Purpose:**

Prompt endovascular treatment of patients with stroke due to intracranial Large Vessel Occlusion (LVO) is a major challenge in rural areas because neurointerventionalists are usually not available. As a result, treatment is delayed, and clinical outcomes are worse compared with patients primarily treated in comprehensive stroke centers (CSC). To address this problem, we present a concept in which interdisciplinary, on-site endovascular treatment is performed in a Primary Stroke Center (PSC) by a team of interventional neuroradiologists and cardiologists: the Rendez-Vous approach.

**Methods:**

Thirty-five patients with LVO who underwent interdisciplinary thrombectomy on-site at the PSC as part of the Rendez-Vous concept were compared with 72 patients who were transferred from a PSCs to the CSC for thrombectomy when diagnosed with LVO in terms of temporal sequences and clinical outcomes.

**Results:**

Patients treated on-site at the PSC as part of the Rendez-Vous approach were managed as successfully and without an increase in complication rates compared with patients treated secondarily at a CSC (91.7% successful interventions in Rendez-Vous vs. 87.3% in control group, *p* = 0.57). The time from diagnosis of LVO to groin puncture was reduced by mean 74.3 min with the Rendez-Vous concept (*p* < 0.01). Regarding the clinical outcome, a functionally independent status was achieved in 45.5% in the Rendez-Vous group and in 22.6% in the control group (*p* = 0.029).

**Conclusion:**

Thanks to interdisciplinary teamwork between cardiology and interventional neuroradiology in local PSCs, times to successful reperfusion can be reduced. This has a potentially positive impact on the clinical outcome of stroke patients.

**Graphical Abstract:**

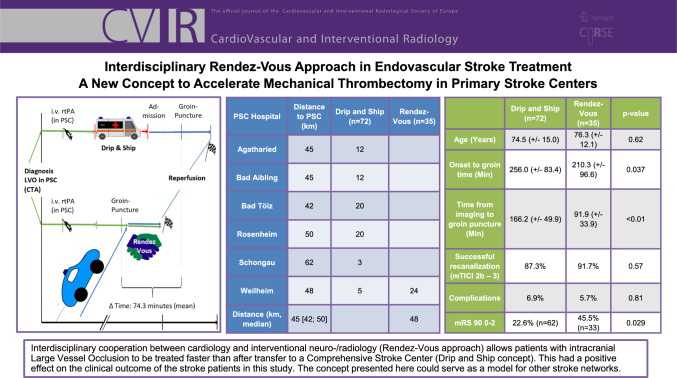

**Supplementary Information:**

The online version contains supplementary material available at 10.1007/s00270-023-03610-y.

## Introduction

Due to the results of Randomized Controlled Trials (RCT), mechanical thrombectomy (MT) has become an elementary component of stroke therapy in the case of Large Vessel Occlusion (LVO) [1–7].

Triggered by the introduction of IVT [8], a dense network of Primary Stroke Centers (PSC), often run by internists (mostly cardiologists) could be established in Germany with the primary aim to initiate IVT as soon as possible [9]. Due to the lack of availability of interventional neuroradiologists, the standard approach to treat patients with LVO is to transfer them to a Comprehensive Stroke Center (CSC) eventually after initiation of IVT (drip-and-ship model). However, this approach consumes considerable time, which has unfavorable impact on patient outcome [10].

In the past years, some centers followed the approach of not transferring the patient to the CSC for MT, but rather bringing the neurointerventionalist to the PSC so that MT can be performed on-site [11–14]. Here, considerable time can be saved [11, 14].

Although the cardiologists cover a wide range of interventional angiographic skills, these skills have so far remained unused in similar projects in the field of interventional stroke therapy [14]. No other medical specialty has such a dense network of acutely available angiography sites including the necessary staff in Germany.

To ensure optimal and fastest possible treatment of stroke patients, we have decided in the concept presented here to cooperate very closely with the specialty of internal medicine, also with regard to endovascular procedures. The special feature of the Rendez-Vous concept is that the internist already starts the procedure of MT before the neuroradiologist is physically present in the partner clinic. Even before the neuroradiologist arrives at the PSC, the main tasks of the on-site team, led by the cardiologist, are to transport the patient to the cardiac catheterization lab, initiate anesthesia unless otherwise discussed, prepare materials, and then also to perform arterial vascular puncture with placement of a sheath and insertion of catheters as arranged with the neuroradiologist by phone. If possible, the cardiologist can also start probing the supra-aortic vessels. Ideally, by the time the neuroradiologist arrives at the PSC, preparations have progressed to the point where only the intracranial thrombectomy itself needs to be performed.

In the current study, we aim to analyze a possible time benefit of the Rendez-Vous approach (intervention group) over the drip-and-ship technique (control group) and, moreover, to compare the technical and clinical outcomes.

## Materials and Methods

For more detailed information about the methods please read supplemental materials.

Inclusion criteria were acutely symptomatic occlusions of the intradural internal carotid artery (ICA), M1-segment of the middle cerebral artery (MCA), proximal M2-segment of the MCA or, occlusion of the basilar artery (BA); see Table 1.

**Table 1:** Baseline characteristics, technical and clinical outcome. MCA, middle cerebral artery; BA, basilar artery; Min, minutes; mTICI, modified thrombolysis in cerebral infarction; mRS, modified Rankin scale; ENT, embolization to new territory; CI, 95% confidence interval The results are indicated either as mean (± standard deviation) or as median with interquartile range [Q1; Q3]

	Drip and Ship (*n* = 72)	Rendez-Vous (*n* = 35)	*P* value
Age (Years)	74.5 (± 15.0)	76.3 (± 12.1)	0.62
Female	61.1%	57.9%	0.80
Intradural ICA	20.8%	21.1%	
MCA (M1, prox. M2)	72.2%	73.7%	
BA	6.9%	5.3%	
Wake-Up Stroke	41.7%	45.8%	0.72
Admission NIHSS	15 [10; 19]	14 [7.5; 18.75]	0.60
Intravenous Thrombolysis	45.8%	40.0%	0.84
**Onset-to-groin time (Min)**	**256.0 (± 83.4)** **236.50 [205;276.75]** CI: (229.32, 282.68)	**210.3 (± 96.6)** **180 [143.75; 253.75]** CI: (166.33, 254.24)	**0.037**
**Time from imaging to groin puncture (Min)**	**166.2 (± 49.9)** **153 [136.5; 183.75]** CI: (154.5, 177.9)	**91.9 (± 33.9)** **91 [72.0; 105.75]** CI: (80.25, 103.5)	** < 0.01**
Duration of intervention	23.5 (± 15.7)	21.4 (± 16.8)	0.81
Number of Maneuvers	2 [1; 3]	1.5 [1; 2]	0.26
Successful recanalization (mTICI 2b – 3)	87.3%	91.7%	0.57
Complications	6.9% 4 × ENT1 x Dissection	5.7%1 × ENT1 x Perforation	0.81
**mRS 90 0–2**	**22.6%** (*n* = 62)	**45.5%** (*n* = 33)	**0.029**

Significant results are displayed in bold

Patients underwent endovascular treatment either in a drip-and-ship concept (control group) in the CSC or on-site in the PSC (Rendez-Vous concept). The two cohorts were compared on the basis of technical and clinical outcome parameters. The study was approved by the local ethics committee.

## Results

The two groups do not differ regarding the patients age, sex, stroke severity (admission NIHSS), occlusion site, and the rate of IVT.

There was no difference regarding interventional treatment success like necessary maneuvers, postinterventional reperfusion, and peri-interventional complications between both groups (see also Table 1). None of the periprocedural complications in the Rendez-Vous group occurred while the cardiologist was alone at the table. The two complications occurred during intracranial thrombectomy by the neuroradiologist, with the cardiologist merely assisting.

**Fig. 1:**
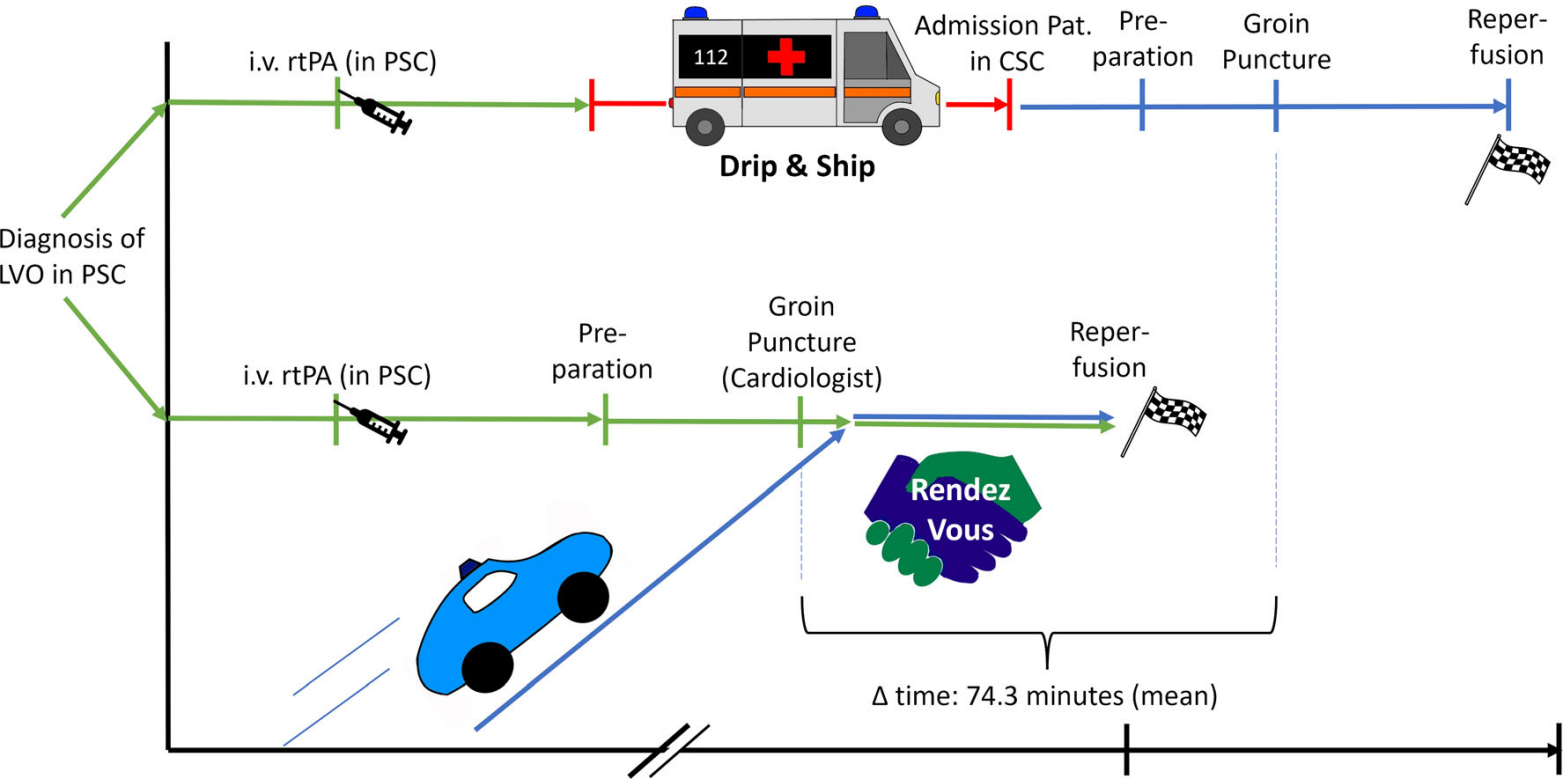
Illustration of the two care concepts compared in the study: "Drip and Ship" model (top) and "Rendez-Vous" concept (bottom). Thanks to the parallelization of the processes, the patient's transport time (red line above) can be eliminated in the Rendez-Vous concept. While the neuroradiologist travels to the PSC in Weilheim, the procedure is prepared on site and the cardiologist already starts the procedure before the neuroradiologist has arrived on site. As a result, the Rendez-Vous approach saved 74.3 minutes (mean) compared to the standard approach from diagnosis to reperfusion.

A major finding was a reduced onset-to-groin time in the Rendez-Vous group (210.3 min vs. 256.0 min, *p* = 0.037), which was achieved by a significantly shorter time from imaging to groin puncture compared with the control group (91.9 vs. 166.2 min, *p* < 0.01), see also Fig. 1. The Rendez-Vous group had a greater proportion of patients with functional independence at 90 days (mRS 90 0–2: 45.5% in Rendez-Vous approach vs. 22.6% in control group, *p* = 0.029), see also Table 1.

## Discussion

The core idea of the Rendez-Vous approach is that interventional cardiology is directly and actively involved in endovascular, interventional stroke procedures. There is a significant time advantage to be gained by active involvement of interventional cardiology, as many on-site activities can be completed by the cardiologist before the (neuro-)radiologist arrives at the scene. Important steps can be taken before the neurointerventionalist arrives.

Not surprisingly, based on previous papers, the technical success rates and periprocedural complications of on-site Rendez-Vous interventions are comparable to those of interventions in the CSC [14, 15]. An important basic requirement is that only experienced interventionalists should be engaged for remote thrombectomies, as these procedures can be more challenging than procedures performed in the familiar setting of the CSC. On the one hand, this concerns the success rate as well as complication management.

Some concern existed at least in Germany among interventional (neuro-)radiologists in the past that interventional stroke care might shift into the area of responsibility of cardiologists. These concerns were mainly based on the similarity of interventional techniques combined with an excellent infrastructure regarding the interventional treatment of myocardial infarction, even in rural areas. In this respect, the interventional cardiologists are busy with cardiological patients in terms of expertise and capacity. An effort to completely take over endovascular stroke treatment is not foreseeable, also with regard to the fact that the number of cases in such primary stroke units is too low for sufficient training including complication management and treatment of very complex cases to take place. To date, stroke medicine in Germany has failed to provide timely on-site thrombectomy in all rural stroke units at all times. However, since MT is a very important treatment modality for ischemic stroke, a suitable concept for each SU must be developed on how to ensure timely MT for patients with LVO. The Rendez-Vous concept could make a contribution for selective PSCs, especially in rural areas. In conjunction with mobile, (neuro-)radiologist thrombectomy services, nationwide coverage of endovascular stroke treatments in rural areas could be further improved. Here, the Rendez-Vous approach could also be integrated into existing mobile stroke services [14] aiming at better outcome for patients suffering severe ischemic stroke caused by LVO.

Our study has several limitations as it is a retrospective approach with small sample size. At this point, we would like to point out a possible selection bias which we cannot completely exclude. Nevertheless, the clinical benefit of the Rendez-Vous concept exceeds the benefit of the Flying Interventionalist concept [16]. The reason for this is not completely clear. However, the “Teleskop” network represents a very special care concept in which daily telemedical or on-site visits take place to be able to provide optimal neurological and neuroradiological support even during the postinterventional course. It is likely that the better outcome is attributable to better postinterventional care. Such a complex care is hardly feasible in large stroke networks with their limited staff and a high number of new teleconsultations every day. It is certainly worth discussing whether health policy makers should acknowledge that the staffing requirements for good telemedicine care are much greater than currently funded.

A total of five patients from Weilheim were included in the control group and were not treated on-site at the PSC as part of the Rendez-Vous concept. In principle, a selection bias could be suspected here. However, three of the five patients had already been treated shortly before the initiation of the Rendez-Vous project, so that an on-site thrombectomy was not possible here. Two additional thrombectomies could not be treated at the PSC for personnel reasons. In one case, the neuroradiologist who was on telestroke duty was acutely ill; in the other case, the cardiac catheterization was occupied by an acute myocardial infarction with coronary intervention. In this respect, we do not consider the data to be significantly biased by these five cases.

Moreover, it cannot be excluded that complications could occur due to the initial activity of the cardiologist, even before the arrival of the neuroradiologist, although this was not the case in the present study. To reduce this risk, the cardiologists were intensively trained in advance about the specific neuroradiological aspects of the procedures. This includes the careful flushing of all inserted catheters. In the Rendez-Vous group analyzed here, in only two cases did the cardiologist have sufficient time before the arrival of the neuroradiologist to begin probing the common carotid artery as an access vessel on his own. Of course, it is conceivable that vascular injuries such as dissections may occur. However, this risk is potentially just as present when neuroradiologists probe these vessels. Ultimately, larger-scale studies will show whether complication rates remain at a low level with this interdisciplinary care concept.

## Conclusion

Interdisciplinary on-site collaboration of interventional (neuro-)radiology and cardiology allows faster cerebral reperfusion therapies in the PSC compared to the standard approach of secondary patient transfers and thrombectomy in the CSC. Supported by close, telemedical, pre- and postinterventional follow-up, a clinical advantage for the Rendez-Vous concept could be demonstrated. This approach might help to further improve stroke care, especially in rural areas.

### Supplementary Information

Below is the link to the electronic supplementary material.

Supplementary file1 (DOCX 20 kb)

## Abbreviations

CSC: Comprehensive Stroke Center
CT: Computed Tomography
DSA: Digital Subtraction Angiography
ENT: Embolization to new territory
EVT: Endovascular treatment
ICA: Internal carotid artery
IVT: Intravenous thrombolysis
LVO: Large vessel occlusion
MT: Mechanical thrombectomy
MR: Magnetic Resonance
NIHSS: National Institutes of Health Stroke Scale
PSC: Primary Stroke Center
RCT: Randomized Controlled Trials
TELESKOP: Telemedizinisches Netzwerk kooperierender Kliniken in Bayern
SU: Stroke Unit
