# Supplementary material for: Interdisciplinary Rendez-Vous Approach in Endovascular Stroke Treatment: A New Concept to Accelerate Mechanical Thrombectomy in Primary Stroke Centers
Source: Cardiovasc Intervent Radiol. 2023 Nov 21;47(1):109–14. doi: 10.1007/s00270-023-03610-y (PMC10769944; doi:10.1007/s00270-023-03610-y)
Supplement: Supplementary file 1 — Supplementary file1 (DOCX 20 kb) [file 270_2023_3610_MOESM1_ESM.docx]

Supplementary Material:

Material and Methods - detailed explanations

This is an observational study. The indications for MT did not differ between the two groups and were in accordance with the current guideline recommendations of the societies of neurology and neuroradiology: acutely symptomatic occlusions of the intradural internal carotid artery (ICA), M1-segment of the middle cerebral artery (MCA), proximal M2-segment of the MCA or, occlusion of the basilar artery (BA); see Table 1. Because the PSC in this study is a telemedicine-managed stroke unit without an on-site 24/7 neurology specialist, all 27 Rendez-Vous patients were evaluated via telemedicine by a neurology specialist from the CSC. Audio-visual data can be transmitted bidirectionally via the telemedicine system (mobile ProCart XL Station Trolley, lean GmbH, Wuppertal, Germany) using a dedicated software tool for telemedical examinations (TrueConf Version 1.1, Zentrum für Telemedizin, ZTM Bad Kissingen, Germany). The telemedicine neurologist can control the camera remotely and zoom in, for example, to examine oculomotor function in high resolution. To a certain extent, the on-site internist acts as an extended arm of the neurologist and performs the examinations according to the neurologist's instructions. The neurologist then determines the National Institutes of Health Stroke Scale (NIHSS).

In any case, the clinical diagnosis of acute ischemic stroke with neurological deficit relevant to daily life had to be present if MT was indicated. For strokes in the anterior circulation, advanced imaging including perfusion imaging had to demonstrate that a relevant mismatch still existed in the extended time window between 6-24 hours. CT perfusion imaging was available 24/7 in both CSC and PSC and was part of the standard diagnostic protocol in both study groups, regardless of the respective time window.

Intervention group/ interdisciplinary Rendez-Vous approach:

All consecutive thirty-five Rendez-Vous interventions between 1/1/2021 and 7/31/2023 presented in this study were performed as part of the telemedical stroke care at Weilheim Hospital, Bavaria, Germany in the “Telemedizinisches Netzwerk kooperierender Kliniken in Bayern” (TELESKOP) network. The TELESKOP network is a telemedical (neuro-)network belonging to the “Neuro-Kopf” Center of the Technical University of Munich. Key services offered in the TELESKOP network to all participating hospitals are the telemedical, neurological patient examination as well as the neuroradiological care of the partner hospitals. The latter includes both diagnostic and interventional services. Furthermore, neurosurgical care is available 24/7 in the TELESKOP network.

Before the Rendez-Vous project could start with the first patient treatment on-site in Weilheim, comprehensive training sessions were held to teach the cardiologists and nursing staff the basics of cerebral MT and to introduce the special, neurointerventional materials and their correct application. The training of the cardiology team consisted of various theoretical and practical training courses and teaching units on site in Weilheim. In addition, several physicians and nurses from the PSC completed internships at the CSC to become fully familiar with state-of-the-art stroke treatments. The internships took place both at the Stroke Unit (SU) and at the Department of Neuroradiology. Active participation in real neuro-angiographies and neuro-interventions were an integral part of the Rendez-Vous curriculum.

All Rendez-Vous procedures in the PSC were performed by an interdisciplinary neuroradiology-cardiology team. The neuroradiology team consisted of four neurointerventional consultants from the Klinikum rechts der Isar, Technical University of Munich and the cardiology team consisted of five consultants from the Weilheim hospital including the nursing cardiac catheterization team, which was also trained separately. For the Rendez-Vous procedures, special angio sets were compiled in advance in sufficient numbers and sterilely packaged so that the cardiological team on site was able to begin preparing the procedure immediately and comprehensively. In addition, all materials and medications needed for MT itself and treatment of any complications that might arise were kept on site in the cardiac catheterization lab. In all Rendez-Vous interventions, treatment was initiated by the cardiologist on site. In all cases, the femoral sheath was successfully placed by the cardiologist and his team on site. In all cases, the actual procedure of mechanical thrombectomy was performed in a lead capacity by the neuroradiologist, whereas the cardiologist acted as a competent assistant during the procedure after transferring the interventional lead role to the neuroradiologist. The angiography system at PSC Weilheim is a Siemens ArtisOne AXN monoplane system that is routinely used for cardiac catheter studies. The system has been upgraded with a special software package for cerebral angiography which also allows high quality neuro-angiography.

Control goup:

Patients with LVO who were transferred from a PSC to our CSC for MT (Drip and Ship patients) were selected for the control group. Typically, these patients did not receive repeat imaging upon arrival at PSC but are taken immediately to angio for intervention. Only in the event of significant changes in clinical presentation was CT/CTA/CTP or MR repeated in the CSC. A five-year observation period was allocated for the study (Aug. 1, 2017-Jan. 8, 2022). Due to their similar distance as Weilheim to the CSC (48 km), transfers from five additional PSCs were included in the "Drip and Ship" control group in addition to the transfers from Weilheim itself (see Table 2). A total of 72 patients consecutive patients who met the inclusion criteria were evaluated as the control cohort. At the CSC, all procedures were performed on a biplane Philips Azurion system. Angiography nurses with 3-12 years of experience in neuro-angiography and cerebral thrombectomy assistance were available at the CSC. In addition, a circulating nurse was available for most of the procedures to sterilely dispense materials.

Angiographic data analysis

All angiographic data were evaluated by two experienced, board-certified neuroradiologists who were not involved in the respective procedures. Evaluation of the modified Thrombolysis in Cerebral Infarction (mTICI) Score [16] as well as assessment of Embolization into New, previously unaffected vascular Territory (ENT) as well as other interventional complications were raised in consensus.

Clinical data evaluation.

Both NIHSS scores and mRS scores were determined by board-certified specialists. The mRS 90 data were collected either by telephone or during face-to-face patient presentations at the CSC for both study groups.

Statistical analysis

Statistical analyses were performed using SPSS software version 28 (IBM, Armonk, NY, USA). Differences between the two groups were compared using the Mann-Whitney U‑test, Fisher’s exact test, or Xi^2^ test, depending on the type of variables analyzed. Statistical significance was assumed at P < 0.05.
